# Recent Progress in Halide Perovskite Nanocrystals for Photocatalytic Hydrogen Evolution

**DOI:** 10.3390/nano13010106

**Published:** 2022-12-25

**Authors:** Zhijie Zhang, Rui Zhou, Deben Li, Ying Jiang, Xuesheng Wang, Huiling Tang, Jiayue Xu

**Affiliations:** School of Materials Science and Engineering, Shanghai Institute of Technology, 100 Haiquan Road, Shanghai 201418, China

**Keywords:** halide perovskite nanocrystals, photocatalysis, hydrogen evolution, heterojunction, charge separation

## Abstract

Due to its environmental cleanliness and high energy density, hydrogen has been deemed as a promising alternative to traditional fossil fuels. Photocatalytic water-splitting using semiconductor materials is a good prospect for hydrogen production in terms of renewable solar energy utilization. In recent years, halide perovskite nanocrystals (NCs) are emerging as a new class of fascinating nanomaterial for light harvesting and photocatalytic applications. This is due to their appealing optoelectronic properties, such as optimal band gaps, high absorption coefficient, high carrier mobility, long carrier diffusion length, etc. In this review, recent progress in halide perovskite NCs for photocatalytic hydrogen evolution is summarized. Emphasis is given to the current strategies that enhance the photocatalytic hydrogen production performance of halide perovskite NCs. Some scientific challenges and perspectives for halide perovskite photocatalysts are also proposed and discussed. It is anticipated that this review will provide valuable references for the future development of halide perovskite-based photocatalysts used in highly efficient hydrogen evolution.

## 1. Introduction

With the rapid growth of human consumption, the world is faced with surging energy demands amidst the quick depletion of fossil fuels and severe natural environmental issues. To alleviate the threat of energy crisis and environmental deterioration, it is urgent to seek more eco-friendly and sustainable renewable energy sources. As a carbon-free and clean energy source, hydrogen has been deemed as a promising future energy source to replace traditional fossil fuels [1,2,3]. Among various approaches to hydrogen generation, photocatalytic water splitting utilizing abundant solar energy and semiconductor materials has been regarded as one of the most attractive routes. In the photocatalysis field, the rational design and fabrication of advanced photocatalysts with ideal solar-to-hydrogen (STH) energy conversion efficiency is the most critical aspect [4,5]. Since the pioneering work of photoelectrochemical water splitting on TiO_2_ electrodes by Fujishima and Honda [6], the field of hydrogen production from water splitting has been systematically investigated, which includes exploring the basic photocatalytic mechanism, developing novel photocatalytic materials, and designing efficient photocatalytic systems. Generally, the fundamental working principle of photocatalysis includes the absorption of light energy by semiconductors to create electron-hole pairs, which then migrate to the semiconductor’s surface to initiate redox reactions. Thus, the separated electron-hole pairs play crucial roles in the photocatalytic redox reaction. To date, various kinds of semiconductor photocatalysts have been developed for hydrogen generation, including oxides (CeO_2_, WO_3_, Ga_2_O_3_, etc.) [7,8,9,10,11,12,13,14,15,16,17], sulfides (CdS, MoS_2_, ZnS, etc.) [18,19,20,21,22,23,24,25,26,27], C_3_N_4_ [28,29,30,31,32,33], etc. However, conventional semiconductor photocatalysts still have a low STH energy conversion efficiency that is far from satisfactory due to their wide bandgaps and high electron-hole recombination rates.

Recently, halide perovskite materials—on account of their fascinating electronic and optical properties, including outstanding visible light harvesting ability, suitable band positions to provide sufficient driving potential, as well as their high carrier mobility and long electron-hole diffusion lengths—have emerged as a class of promising candidates for photocatalytic applications. To date, various kinds of halide perovskites, in either an organic–inorganic or all-inorganic fashion (e.g., CH_3_NH_3_PbX_3_, CsPbX_3_, X = Cl, Br, I), have shown great potential in photocatalysis fields such as CO_2_ reduction [34,35,36,37,38,39,40], hydrogen generation [41,42,43,44,45], pollutant degradation [46,47,48,49,50,51], phemethylol oxidation [52,53], organic reaction [54,55], etc. Since the first demonstration of using methylammonium lead iodide (CH_3_NH_3_PbI_3_, MAPbI_3_) for hydrogen generation via the solar-driven splitting of hydrogen iodide by Park et al. [44], the potential of halide perovskites for photocatalytic hydrogen production has been investigated by many researchers. In virtue of the advantages of halide perovskites mentioned above, such as the excellent light-absorption ability and suitable band positions, the applications of these materials in photocatalytic hydrogen production begin to flourish and exhibit outstanding photocatalytic performances.

In this review, we summarize the recent progress made in using halide perovskites for solar-driven photocatalytic hydrogen production. To date, numerous review articles have summarized recent advances in halide perovskites applied in solar energy conversion. In contrast to previous reviews, this review focuses exclusively on the application of halide perovskites in photocatalytic hydrogen production. Firstly, we introduced the property and photocatalytic mechanism of halide perovskites, and highlighted strategies used for enhancing the photocatalytic hydrogen production performance of these materials. Finally, we concluded by introducing a perspective on the future challenges and opportunities of this field, which could provide guidelines for further research on halide perovskite-based photocatalysis applications. 

## 2. Properties of Halide Perovskites

### 2.1. The Composition of Halide Perovskites 

**Figure 1:**
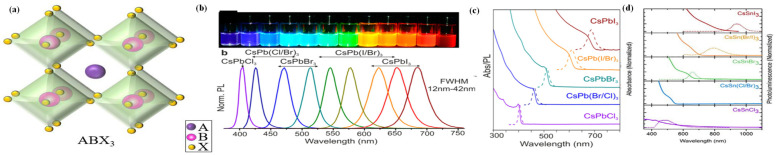
(**a**) Schematic illustration of the crystal structure of halide perovskite. (**b**) Colloidal halide perovskite CsPbX_3_ NCs (X = Cl, Br, and I) exhibit size- and composition-tunable bandgap energies covering the entire visible spectral region with narrow and bright emission. (**c**) Typical optical absorption and PL spectra of CsPbX_3_ NCs. (**d**) Typical optical absorption and PL spectra of CsSnX_3_ NCs. (**b**,**c**) Reproduced with permission [70]. Copyright: 2015, American Chemical Society. (**d**) Reproduced with permission [71]. Copyright: 2016, American Chemical Society.

Halide perovskite materials have a general structural formula of ABX_3_ (Figure 1a), where A is usually a monovalent cation (Cs^+^, methylammonium (MA^+^), or formamidine (FA^+^)), B is a divalent metal ion (Pb^2+^, Sn^2+^, or Ge^2+^), and X is the halogen anion, namely I^−^, Br^−^, or Cl^−^ [56,57]. The B cation coordinates with six halogen anions to form [BX_6_]^4−^ octahedra. The large monovalent A cation behaves like a total charge neutralizer, which is bridged to a network of corner-sharing BX_6_ octahedrons, forming the ideal perovskite structure. The A-, B-, and C-site ions can be substituted isomorphically by other similar ions, thus altering the defect properties, electronic structure, and catalytic performance of the material [58,59,60,61]. Both the B and X ions play an important part in governing the band structure of halide perovskites that affects their catalytic performance, while the function of the A cation is ignorable. Although the A cation generally does not construct the energy level, its size plays a crucial role in judging the formability of the perovskite structure, since either a smaller or larger A cation could lead to either a contraction or expansion of the perovskite lattice. Usually, perovskites exhibit a cubic structure (space group: Pm3m), which can transform into orthorhombic (space group: Pnma) or tetragonal (space group: I4/mcm) phase when the temperature decreases [62,63,64]. As observed in the crystal structure, the B-site cation and the anion are tightly bound, while the A-site cation and the anion have a weak interaction. The BX_6_ octahedral structure can be distorted by the difference between the electronegativities and ionic radii of the A and B cations, leading to a weakened symmetry of the crystal structure. It has been proved that the tilt angle can affect the electronic band structure, photoluminescence, dielectric, and the charge transport properties of the perovskites [65,66,67]. Various types of perovskite crystals with desirable characteristics can be designed by adjusting these interactions via placing different types of cations at corresponding lattice sites [68,69].

### 2.2. Optoelectronic Properties of Halide Perovskites 

Understanding the composition, crystal structure, and the electronic band structure of the perovskite is of vital importance because these pivotal factors are intercorrelated in judging its potentiality for satisfactory photocatalytic performances. The energy band strucure of halide perovskites consists of an antibonding valence band maximum (VBM) and an antibonding conduction band minimum (CBM) system, rendering this crystal with high defect tolerance. Applying DFT calculations, the VBM of perovskites is composed of an antibonding hybrid state between the 6s orbital of B and the np orbital of X (n = 3, 4, and 5 for Cl, Br, and I, respectively), with the np orbital of X as the major contributor, whereas the CBM is formed from an antibonding hybrid state between 6p orbitals of B and np orbitals of X, with 6p orbitals of B as the significant contributor [72,73]. Taking cubic CsPbBr_3_ QDs as an example, its CB and VB positions are determined based on the 6p orbital of Pb and the 4p orbital of Br, respectively, whereas Cs has negligible effect on the energy band edge [74]. Since the A-site cations exhibit no significant effect on the VBM or CBM [75], the bandgap tuning can be easily realized by mixing or exchanging the halogen anions. For example, by altering the ratio of different halide ions, Sargent and his co-workers successfully tuned the bandgap of MAPbI_3_ [76]. When the iodide concentration was increased, the absorption and emission spectra of the CsPbBr_3−x_I_x_ perovskite film red-shifted to longer wavelength, and its bandgap became narrower [77]. The tunable bandgap affords a good platform to modulate the energy band edge of halide perovskites toward highly efficient photocatalytic performance for various applications. For instance, Guo et al. reported that the band edge positions of CsPb(Br_x_/Cl_1−x_)_3_ could be tuned by regulating the ratio of Br and Cl [78], and the photocatalytic activity toward CO_2_ reduction is significantly enhanced.

Generally, halide perovskites are considered as direct bandgap semiconductors. For MAPbI_3_, the spin–orbit coupling leads to Rashba splitting of the conduction band, resulting in a weak indirect bandgap of 60 meV during the direct bandgap transition [79]. Halide perovskites exhibit photo-absorption in almost the entire visible region, indicating that charge carriers can be effectively produced upon low energy excitation, which is favourable to photocatalytic applications [80,81]. A practical strategy to realize spectral absorption diversity and bandgap tuning with perovskites is adopting mixed halides. For example, by only altering the halogen element at the X-site from Cl to Br to I, Protesescu et al. found a redshift of the emission wavelength of CsPbX_3_ (X = Cl, Br, or I) from 410 nm to 512 nm and 700 nm, and to any other intermediate wavelengths within the visible spectral range using mixed halide ions (Figure 1b,c) [70]. However, as the halogen composition changes, the valence band edge moves by a relatively wide margin within the energy levels, while the conduction band edge exhibits little change. For the MAPbX_3_ perovskite, when X was Cl to I, the emission wavelength shifted from 403 to 740 nm [82].

In addition, the B-site cation also has a significant effect on the optical properties of halide perovskites. Inevitably, the Pb element in perovskites need to be partially or completely replaced due to environmental issues. Compared to those of CsPbX_3_, strong red shifts of the absorption and emission spectra were observed for CsSnX_3_, from 443 nm (X = Cl) to 953 (X = I) (Figure 1d) [71]. By compositional modulation, the bandgaps of halide perovskites can be designed and tuned within a certain range, achieving targeted energy levels. In addition, optical properties of halide perovskites can be enhanced by a metal ion doping strategy. For example, by doping Cu^2+^ and Sb^3+^ into the three octahedral layers, the layered double perovskite Cs_4_CuSb_2_Cl_12_ was obtained. It has a direct band gap of 1.0 eV, and an electrical conductivity one order of magnitude higher than that of MAPbI_3_ [83]. In addition, the partial replacement of Pb by Mn may cause a strong Stokes shift in the emission, which can increase the utilization rate of sunlight [84]. However, the incorporation of cations (Cd^2+^, Al^2+^, and Zn^2+^) into the halide perovskite can cause shrinkage of the original lattice, resulting in a wider bandgap, blueshift of the absorption peak, and weaker absorption ability [85,86,87].

## 3. Applications of Halide Perovskites in Photocatalytic Hydrogen Evolution

### 3.1. Basic Principle of Photocatalysis with Halide Perovskites

**Figure 2:**
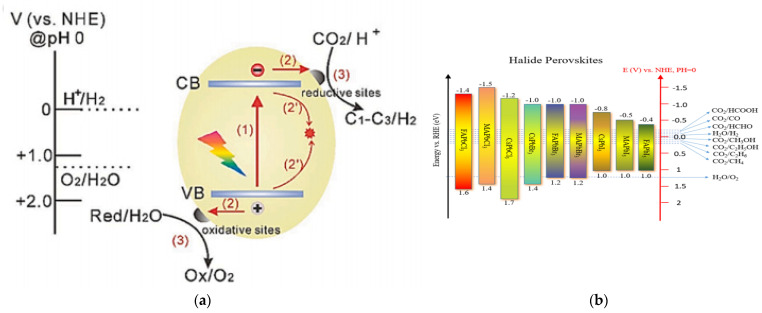
(**a**) Schematic illustration of charge transfer reactions that may occur at the surface and in the bulk of a semiconductor photocatalyst. (**b**) Energy levels of halide perovskites with the relative potential in photocatalytic applications. (**a**) Reproduced with permission [88]. Copyright: 2020, Wiley.

Photocatalytic redox reactions driven by semiconductor materials usually involve identically essential steps (Figure 2a) [88]: (1) generation of electron-hole pairs by the light harvesting of the photocatalyst, (2) transfer of photogenerated electrons and holes to the surface of the photocatalyst, and (3) photogenerated charge carriers participate in redox reactions. In order to drive the water-splitting redox reactions, the VB edge of the semiconductor should be more positive than the oxidation potential of H_2_O to O_2_ (1.23 V vs. normal hydrogen electrode [NHE], pH = 0), while the CB edge of the semiconductor should be more negative than the reduction potential of H^+^ to H_2_ (0 V vs. NHE, pH = 0) [89]. So, theoretically, the minimum bandgap required for water splitting is 1.23 eV. However, considering the overpotential associated with the water-splitting redox reactions, the bandgap to drive efficient overall water splitting must be further widened, usually to 1.8–2.0 eV [90,91,92]. The relative positions of CB and VB potentials for most halide perovskites are shown in Figure 2b, along with the redox potentials of photocatalytic half-reactions associated with water splitting, CO_2_ reduction, etc. Apparently, the CB potentials of most halide perovskites are more negative than the reduction potential of H^+^ to H_2_, meeting the thermodynamic requirements for reducing water. In other words, the relative CB positions of halide perovskites are usually sufficient for H_2_ production, exhibiting excellent reduction abilities. Theoretically, some members of halide perovskites (such as all-inorganic CsPbBr_3_) can also oxidize water to produce O_2_ because their VB potentials are relatively positive. 

In addition, factors like the molar extinction coefficient, charge carrier recombination, and defect state of a halide perovskite should also be taken into account for photocatalytic applications [93,94,95]. A high molar extinction coefficient (ε) is essential for efficient absorption of visible light and generation of excitons [96]. The ε values of colloidal perovskites, ranging from about 10^5^ to 10^7^ L mol^−1^ cm^−1^, are comparable to those of a-Si: H and GaAs, and an order of magnitude higher than that of c-Si, which are representative photovoltaic materials [97,98]. This is indicative of better visible light responses for halide perovskites, thus improving photon-carrier conversion efficiencies [99]. However, the ε value of a halide perovskite is also dependent on the size of the crystals. This is especially important in the nanometer size range due to the quantification effect, which needs to be taken into account for practical applications in optoelectronics [100]. 

Carrier diffusion length is also important for the photocatalytic applications of halide perovskites. Generally, a longer carrier diffusion length indicates a lower recombination rate. The carrier diffusion lengths of halide perovskites have been increased by various strategies. For example, Dong et al. used a solution–growth method to obtain MAPbI_3_ single crystals with a high carrier diffusion length exceeding 175 μm, which could be attributed to the long lifetime, high carrier mobility, and small trap densities of the single crystals [101], whereas the polycrystalline MAPbI_3_ has a charge carrier diffusion of only ca. 100 nm. By incorporating Cl^−^ into MAPbI_3−x_Cl_x_, Zhang et al. obtained a perovskite with a carrier diffusion length of up to 380 μm [102]. This is because the incorporation of Cl^−^ can increase the density of trap states, thus creating the medium for carrier transfer and reducing the VB, which play a dominate role in charge recombination. As a consequence, maximum values of the carrier diffusion lengths were reached for the MAPbI_3−x_Cl_x_ single crystals with the optimum Cl content (x = 0.005). Owing to the enhanced utilization of photogenerated charge carriers, it is expected that the long carrier diffusion length of halide perovskites would significantly contribute to their catalytic activity.

In addition, the photogenerated charge transfer kinetics in halide perovskites are also vital for the design of highly efficient photocatalysts. Excitons and free carriers are produced rapidly under light excitation, with free carriers as the main light-excited species. In most cases, excitons are also rapidly decomposed into free carriers. For instance, the excitons in CsPbBr_3_ NCs are quickly converted into free carriers after 4–5 ps [103]. These hot carriers relax to the energy band edge via carrier–phonon and carrier–carrier interactions within fs. The larger the size of halide perovskite nanocrystals is, the faster the cooling kinetics of hot carriers is [104]. As the lifetime of a hot carrier in perovskites increases, the carrier becomes relatively easy to extract. Radiative recombination of cooled carriers takes place within ns at the band edge. For example, the capture time of non-radiative carriers in CsPbBr_3_ NCs is about 40–50 ps [105]. On account of the defect tolerance features of halide perovskites, the energies of carriers in the defects are similar to those of edge carriers, which implies that more high-energy carriers will make potential contributions to photocatalytic reactions. For CsPbBr_3_, the carriers can be extracted by electron-hole acceptors within ps, indicating that the extracted carriers can be potentially applied in photocatalysis [106]. Therefore, the ideal energy levels, along with the unique charge transfer kinetics, make halide perovskites good candidates for photocatalytic applications [107]. To sum up, halide perovskites provide distinct advantages for photocatalysis: (1) targeted electronic structures can be designed by altering the A, B, and X-site elements in the crystal structure, so that other physical properties can be tailored, such as stability, light absorption, and charge migration [108]; (2) the unique energy band structures endow halide perovskites with suitable band edge positions to drive a broad range of photocatalytic reactions [109]; (3) the long carrier diffusion lengths and high charge mobility also render halide perovskites as promising candidates for the design of high-performance photocatalysts.

### 3.2. Halide Perovskites for Hydrogen Evolution

#### 3.2.1. Pristine Halide Perovskites or Solid Solutions

**Figure 3:**
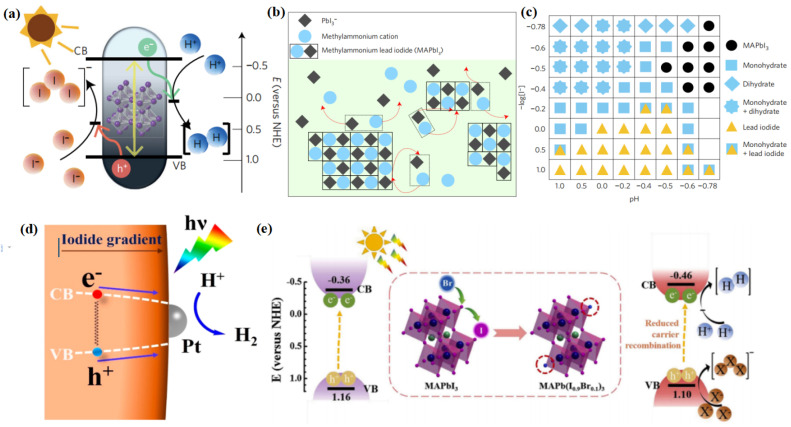
(**a**) Schematic energy band structure of MAPbI_3_ powder for the photocatalytic HI splitting reaction. (**b**) Schematic illustration of the MAPbI_3_ powder in dynamic equilibrium in saturated HI solution. The red color arrows represent dissociation and reprecipitation of MAPbI_3_ crystal and ions. (**c**) Constructed phase map as a function of [I^−^] and [H^+^]. Each symbol represents the stable precipitate phases in saturated solutions at each [I^−^] and [H^+^] concentration. Main peaks of precipitated powder are not indexed under some conditions, expressed as empty boxes. (**d**) Promoted charge separation and enhanced photocatalytic H_2_ evolution by formation of a bandgap funnel structure of MAPbBr_3−x_I_x_ near the surface. (**e**) Schematic band diagram of MAPbI_3_ and MAPb(I_1−x_Br_x_)_3_ (x = 0.10) crystal for photocatalytic HI splitting reaction. (**a**–**c**) Reproduced with permission [44]. Copyright: 2016, Nature Publishing Group. (**d**) Reproduced with permission [110]. Copyright: 2018, American Chemical Society. (**e**) Reproduced with permission [111]. Copyright: 2019, Elsevier.

As discussed above, the unique optoelectronic properties of halide perovskites are favourable to their application in photocatalytic reactions, including hydrogen evolution. The first milestone for photocatalytic hydrogen evolution using halide perovskites was reported by Park et al. [44]. As is known, most halide perovskites are unstable in polar solvents, especially water [87]. In order to conquer this, Park and his coworkers employed the dynamically balanced HI solution as the reaction medium, which can maintain the stability of MAPbI_3_ (Figure 3a). MAPbI_3_ is regarded as an ionic crystal consisting of MA^+^ and PbI_3_^−^, which can be precipitated in saturated solutions. Hence, when MAPbI_3_ precipitates are dissolved in saturated solution, they can be decomposed into MA^+^ and PbI_3_^−^ ions. Simultaneously, MA^+^ and PbI_3_^−^ ions were reprecipitated into crystals at the same rate. In this way, the MAPbI_3_ powder could maintain stability in aqueous HI solution ((Figure 3b). They also found out that different phases of MAPbI_3_ can exist, depending on the concentrations of H^+^ and I^−^. However, only under the specific conditions of [I^−^] ≤ [H^+^], pH < −0.5, and −log [I^−^] < −0.4 can MAPbI_3_ remain stable (Figure 3c). This pioneering work has opened up a way for using halide perovskites in photocatalytic fields. Afterward, Wang and his group elucidated the reaction mechanism of photocatalytic hydrogen evolution using MAPbI_3_ [45]. In this reaction, MAPbI_3_ played dual roles as a visible light photoabsorber and as a catalyst reductant. Meanwhile, both the Pb atoms and surface organic molecules participated in the reaction. First, an intermediate state of Pb—H was formed by the interaction between one H atom dissociated from an MA^+^ ion and Pb. Subsequently, H_2_ was produced by the reaction of the Pb—H intermediate state with another H atom from an adjacent MA^+^ ion. The lost H would be replaced by protons from the solution to produce new MA^+^ ions through the Grotthuss mechanism.

As mentioned above, the CBM and VBM potentials of halide perovskites can be modified by modulating their compositions, which makes them suitable for gradient photocatalysis. By tuning the iodide concentration gradient, Huang et al. synthesized a mixed halide perovskite material (MAPbBr_3−x_I_x_) with a funnel-like bandgap structure [110]. The CBM becomes more positive with the increase of I concentration from the interior to the surface, whereas the VBM becomes more negative (Figure 3d). In this way, a smooth funnel is constructed, which promoted the charge transfer from the inside to the surface. As a consequence, this specially designed photocatalyst exhibited a H_2_ generation rate of 255.3 μmol h^−1^. When Pt was further loaded on the surface of the perovskite, the photogenerated electrons on the perovskite surface transferred rapidly to the Pt particles, which further increased the H_2_ generation rate to 651.2 μmol h^−1^. In a similar fashion, Huang’s group also constructed a bandgap funnel-structured CsPbBr_3−x_I_x_ mixed halide perovskite via the graded distribution of iodide [112]. The obtained CsPbBr_3−x_I_x_/Pt photocatalysts exhibited a H_2_ evolution rate of 1120 μmol g^−1^ h^−1^ under visible light irradiation, along with a high stability during the 50 h of the photocatalytic experiment.

Considering the stability issues of halide perovskites, photocatalytic hydrogen generation using these materials are often conducted in HX (X = Cl, Br, or I) solution instead of direct water splitting [113]. Water splitting is a four-electron reaction, while the reduction of HI involves two electrons. When electrons drive the H_2_ production reaction, I_3_^−^ is generated during the photocatalytic process, which will darken the reaction medium gradually. As a consequence, the light absorption of the photocatalyst will be interfered. This can be overcome by the addition of hypophosphorus acid (H_3_PO_2_) as a chemical stabilizer, which can maintain the concentration of I^−^ and reduce the I_3_^−^ ions [110]. Doping Br ions have also proved an effective way to enhance the photocatalytic HI splitting activity of MAPbI_3_ [111]. The resultant MAPb(I_1−x_Br_x_)_3_ perovskite exhibited a high H_2_ evolution of 1471 μmol h^−1^ g^−1^ even without a Pt cocatalyst. This is because the addition of Br ions can tune the band structure of perovskite, with a negative shift of the CB potential, thus enhancing the reduction capability of electrons for efficient H_2_ production (Figure 3e). In addition, the Br-incorporated perovskite has a lower H-Pb absorption energy, which makes it easier for H to transfer from MA^+^ to the Pb atom at the defect site, thus increasing the H_2_ evolution rate.

#### 3.2.2. Halide Perovskite Composites

**Figure 4:**
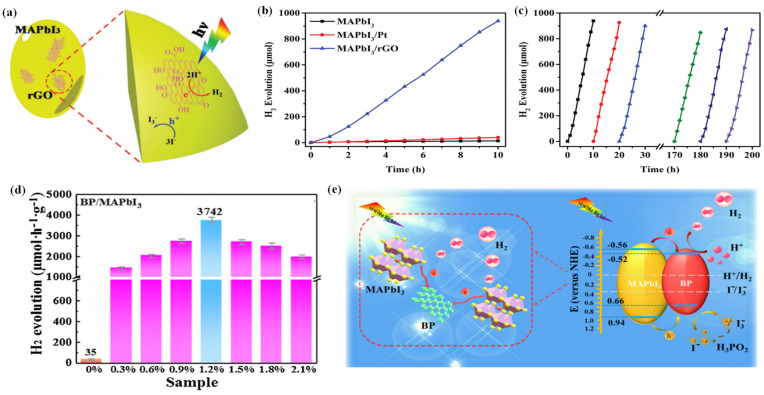
(**a**) Schematic illustration of photocatalytic H_2_ evolution by MAPbI_3_/rGO. (**b**) Comparison of the H_2_ evolution performance of MAPbI_3_, MAPbI_3_/Pt, and MAPbI_3_/rGO. (**c**) Stability test of MAPbI_3_/rGO during 20 cycles of H_2_ evolution experiments. Lines with different colors represent different cycles. (**d**) Photocatalytic H_2_ evolution rates of BP/MAPbI_3_. (**e**) Schematic mechanism of the photogenerated charge transfer in the BP/MAPbI_3_ composite under visible light irradiation. (**a**–**c**) Reproduced with permission [41]. Copyright: 2018, Wiley. (**d**,**e**) Reproduced with permission [114]. Copyright: 2019, Elsevier.

Wu et al. reported a MAPbI_3_/rGO composite with outstanding photocatalytic performance in aqueous HI solution (Figure 4a) [41]. It has a high H_2_ evolution rate of 93.9 µmol h^−1^ under visible light irradiation, which is 67 times and 23 times higher than that of pristine MAPbI_3_ and Pt-loaded MAPbI_3_, respectively (Figure 4b). The remarkable performance could be attributed to the introduction of rGO, which possesses good charge transport ability and facilitates charge transfer. The electrons that transfer from MAPbI_3_ to rGO then reduce protons to H_2_, resulting in excellent photocatalytic activity of the MAPbI_3_/rGO composite. Moreover, the composite is extremely stable, with no significant decrease of the H_2_ evolution activity after 200 h of cyclic experiments (Figure 4c). As confirmed by XRD, the recycled photocatalyst showed no change or failure in structure. That was because the MAPbI_3_ powders and the saturated HI solution were in dynamic equilibrium. When the reaction occurred, the exposed MAPbI_3_ surface was restored all the time, ensuring the continuous oxidation of I^−^ to I_3_^−^ on the surface in contact with HI.

Li et al. anchored a 2D few-layer black phosphorus (BP) on MAPbI_3_ via electrostatic coupling and fabricated a BP/MAPbI_3_ composite for photocatalytic hydrogen evolution [114]. The resultant BP/MAPbI_3_ exhibited a superb photocatalytic hydrogen evolution rate of 3742 μmol h^−1^ g^−1^ under visible light, which was far higher than that of both pure MAPbI_3_ and MAPbI_3_/Pt (Figure 4d). Moreover, the BP/MAPbI_3_ showed superior durability without no obvious decrease in the activity after 20 cycles. The outstanding photocatalytic activity and stability of the BP/MAPbI_3_ could be attributed to the broadened light harvesting, enhanced charge separation, and high chemical/optical stability of BP/MAPbI_3_ composite in HI solution (Figure 4e).

**Figure 5:**
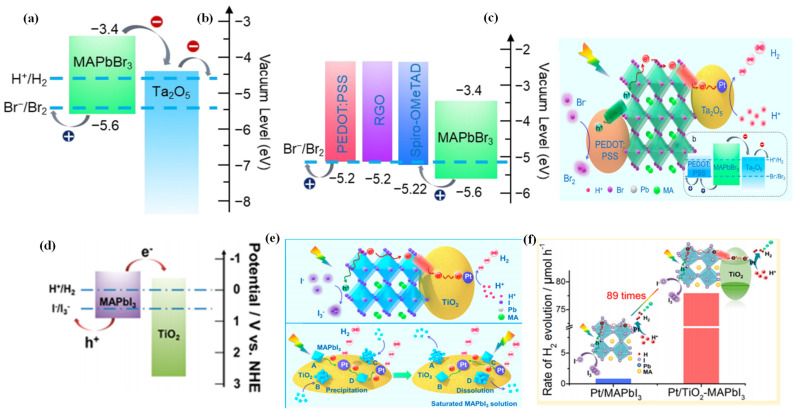
(**a**) Schematic energy levels of MAPbBr_3_ and Ta_2_O_5_ and the redox potentials for HBr splitting reaction. (**b**) Energy level diagrams of MAPbBr_3_ and hole-transporting materials. (**c**) Schematic illustration of the reaction mechanism for MAPbBr_3_ with Pt/Ta_2_O_5_ and PEDOT:PSS as the electron- and hole-transporting channels, respectively. (**d**) Schematic diagrams of energy band of MAPbI_3_ and TiO_2_. (**e**) Schematic illustration of photocatalytic HI splitting for H_2_ evolution by Pt/TiO_2_-MAPbI_3_ hybrid system under visible light irradiation. (**f**) Comparison of H_2_ evolution activity over Pt/MAPbI_3_ and Pt/TiO_2_-MAPbI_3_. (**a**–**c**) Reproduced with permission [115]. Copyright: 2019, American Chemical Society. (**d**–**f**) Reproduced with permission [42]. Copyright: 2018, American Chemical Society.

Wang et al. adopted a novel simultaneous dual-charge transportation modulation approach to improve the photocatalytic H_2_ evolution activity of organic–inorganic MAPbBr_3_ NCs [115]. They hybridized the MAPbBr_3_ perovskite with Pt/Ta_2_O_5_ and poly(3,-4-ethylenedioxythiophene):polystyrenesulfonate (PEDOT:PSS) nanoparticles, which acted as electron- and hole-transporting motifs, respectively. By providing new dual-charge transporting pathways, the charge separation and transportation efficiency of MAPbBr_3_ was significantly improved. Tantalum pentoxide (Ta_2_O_5_) was selected for its ideal conduction band edge position, which can provide an electron transport pathway to accelerate the electron transportation from MAPbBr_3_ (Figure 5a). Thus, Pt/Ta_2_O_5_-MAPbBr_3_ contributed to the increase of H_2_ evolution rate. PEDOT:PSS was used as an efficient hole-transporting material in the hybrid system for its more positive VBM than that of MAPbBr_3_, which facilitated the Br-oxidation reaction (Figure 5b). Therefore, Pt/Ta_2_O_5_-MAPbBr_3_-PEDOT:PSS was the most effective photocatalyst for H_2_ evolution (Figure 5c). The photocatalytic hydrogen evolution rate on the hybridized system was increased by ca. 52 times than that of pristine MAPbBr_3_, with an apparent quantum efficiency up to 16.4% at 420 nm.

Through hybridization of MAPbI_3_ with Pt/TiO_2_, Wang et al. greatly enhanced the photocatalytic hydrogen evolution rate of MAPbI_3_ from HI splitting [42]. Due to the suitable band alignment (Figure 5d), the TiO_2_ nanoparticles can act as nanoscale electron-transporting channels, which allow efficient extraction of the photogenerated electrons from MAPbI_3_. As illustrated in Figure 5e, the introduction of Pt/TiO_2_ could create dynamically existing electron-transporting channels between the MAPbI_3_ and Pt/TiO_2_, which drastically enhanced the charge transportation efficiency of MAPbI_3_ nanoparticles. As a consequence, the photocatalytic hydrogen evolution rate of Pt/TiO_2_-MAPbI_3_ was enhanced by ca. 89 times than that of Pt/MAPbI_3_ (Figure 5f).

Wang et al. employed MoS_2_ nanosheets as a cocatalyst to couple with MAPbI_3_ and fabricated a MAPbI_3_/MoS_2_ composite for photocatalytic H_2_ evolution [116]. Since the conduction band potential of MAPbI_3_ is more negative than that of MoS_2_, the photogenerated electrons can efficiently transfer from MAPbI_3_ to MoS_2_, which hindered the carrier recombination rates. As a result, the MAPbI_3_/MoS_2_ composite exhibited a H_2_ evolution rate 121 times higher than pristine MAPbI_3_.

#### 3.2.3. Pb-Free Halide Perovskites

In order to overcome the toxicity of lead, Guo et al. developed an eco-friendly lead-free perovskite MA_3_Bi_2_I_9_ and applied it for photocatalytic H_2_ evolution [117]. Owing to the precipitation–solubility equilibrium reached in the system, the obtained MA_3_Bi_2_I_9_ exhibited excellent phase stability in HI solution. After 70 h of repeated H_2_ evolution, it showed no degradation or oxidization with satisfactory cycle stability. When using Pt as a cocatalyst, the H_2_ production rate was enhanced by 14 times compared with the pristine one, reaching 169.21 μmol g^−1^ h^−1^.

**Figure 6:**
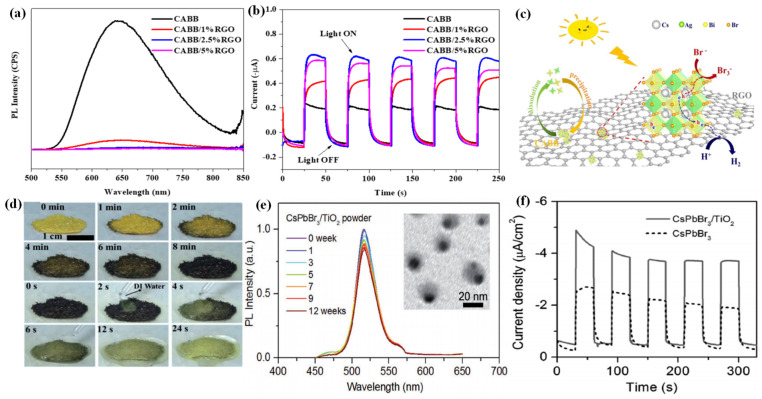
(**a**) Steady-state PL spectra of the CABB/xRGO composites (x = 0, 1%, 2.5%, 5%). (**b**) Photocurrent responses of the CABB/xRGO samples (x = 0, 1%, 2.5%, 5%) recorded at 0 V vs. Ag/AgCl electrode. (**c**) Schematic mechanism of photocatalytic HBr splitting by CABB/RGO under visible light irradiation. (**d**) In situ observation of the reversible DMASnI_3_ transformation process at 80 °C in air. (**e**) The relative PL intensity of CsPbBr_3_/TiO_2_ NCs after immersing in Milli-Q water (0–12 weeks). Inset: TEM image of CsPbBr_3_/TiO_2_ NCs after immersing in Millil-Q water for 12 weeks. (**f**) Transient photocurrent responses of CsPbBr_3_ and CsPbBr_3_/TiO_2_ NCs electrodes at −0.1 V versus NHE. (**a**–**c**) Reproduced with permission [118]. Copyright: 2020, Elsevier. (**d**) Reproduced with permission [119]. Copyright: 2018, Wiley. (**e**,**f**) Reproduced with permission [120]. Copyright: 2018, Wiley.

For the first time, Zhao et al. applied the Cs_2_AgBiBr_6_ (CABB) double perovskite for HBr splitting under visible light irradiation, in which RGO was introduced to extract the photogenerated electrons from CABB [118]. The resultant CABB/RGO composite exhibited a H_2_ evolution of 489 μmol g^−1^ within 10 h under visible light irradiation, which was 80 times higher than that of bare CABB. Moreover, the CABB/RGO composite with optimal RGO demonstrated ideal stability, with no significant decline in H_2_ evolution after 120 h continuous photocatalytic reaction. As confirmed by the photoluminescence (PL) (Figure 6a) and photoelectrochemical measurements (Figure 6b), the CABB/RGO composite exhibited suppressed charge recombination and better charge transfer ability than bare CABB. This could be attributed to the introduction of conductive RGO, which could accelerate the electron transfer from CABB through the M-O-C bonds. Subsequently, the transfered electrons reduce H^+^ to generate H_2_ at the active sites of RGO, while the holes on CABB particles oxidized Br- to produce Br_3_^−^ (Figure 6c).

#### 3.2.4. Water Stable Halide Perovskites

In order to overcome the stability issues of most halide perovskites, Ju et al. developed a lead-free hybrid perovskite single-crystal DMASnI_3_ (DMA = CH_3_NH_2_CH^+^) with excellent water phase stability [119]. No decomposition was observed when DMASnI_3_ was immersed in deionized water for 16 h. Inspired by this, they applied DMASnI_3_ as a photocatalyst for H_2_ evolution in deionized water. A H_2_ evolution rate of 0.64 µmol h^−1^ was observed on the DMASnI_3_ crystals, accompanied by good recycling properties. Interestingly, the DMASnI_3_ crystals exhibited a reversible band gap narrowing behavior without phase transformation. When exposed to deionized water, the transformed samples in black can self-heal into yellow ones rapidly (Figure 6d). The narrow band gap, high stability, as well as outstanding electrical properties render DMASnI_3_ as a promising optoelectronic material. By the encapsulation of colloidal CsPbBr_3_ NCs into the TiO_2_ shell, Li et al. obtained nearly monodispersed CsPbBr_3_/TiO_2_ core/shell NCs with excellent water stability [120]. The size, structure, morphology, and optical properties remained identical after the CsPbBr_3_/TiO_2_ core/shell NCs were immersed in water for three months (Figure 6e), representing one of the most water-stable inorganic shell passivated perovskite NCs. Moreover, owing to the electrical conductivity of the TiO_2_ shell, the CsPbBr_3_/TiO_2_ core/shell NCs exhibited increased charge separation efficiency (Figure 6f), making it a potential material for optoelectronic and photocatalytic applications in aqueous phase. The photocatalytic performance of halide perovskite-based systems for hydrogen generation is summarized in Table 1.

## 4. Conclusions and Prospects

In this review, we have introduced the recent advances made in the field of halide perovskite-based hydrogen evolution, focusing on the strategies to enhance the photocatalytic activity of these materials. Although halide perovskites have intriguing properties, their poor stabilities arising from the soft ionic crystal structures restrict their application in photocatalysis. However, via intrinsically improving the crystal stabilities of halide perovskites, several “stable” photocatalytic systems based on these kind of materials have been designed [119,120]. Moreover, by altering the external reaction conditions, such as using saturated halo acid solutions as the solvent, halide perovskites have been successfully used in photocatalytic hydrogen evolution [41,42,44,114,115,116,117,118,119,121,122,123,124,125,126,127,128]. In spite of this, there are still big challenges when applying halide perovskites in photocatalytic reactions under more common environments. On the basis of the current level of knowledge and the limitations of halide perovskites, some promising approaches to enhance the activity and stability of halide perovskite-based photocatalysis are proposed.

### 4.1. Improving the Long-Term Catalytic Stability of Halide Perovskites

Although conducting photocatalytic reactions in halo acid solutions have been proven effective for hydrogen evolution, such methods are not universal in nature. To limit contact between halide perovskites and the polar solvent, it is essential to explore excellent sealing technology to secure the stability of halide perovskites. There are two essential criteria to be considered when establishing this technology: one is the transparency of the sealing materials which can ensure sufficient light absorption of halide perovskites. The second criterion is the good conductivity of the sealing material that can allow the effective extraction of photo-generated carriers. At present, transparent resin (epoxy) is the most commonly used sealing material. Conductive carbon paste is also employed as the sealing agent in some studies, considering its conductive property and higher resistance towards degradation. Alternatively, some researchers encapsulate halide perovskites with electron- and hole-transport materials together to fabricate corresponding photoelectrodes for photocatalysis. In addition, halide perovskites can be separated from the polar solvent by encapsulating the perovskite layer in a solar cell structure to develop a PV–PEC reaction system. This system has the advantage of increased redox capacity of the PEC cell owing to the photovoltaic device, which allows larger voltage in series and thus supporting a wide range of applications.

### 4.2. Improving the H_2_ Generation Activity of Halide Perovskite-Based Photocatalysts

As shown in Table 1, the highest H_2_ evolution rate so far achieved for halide perovskite-based photocatalyst is about 13.6 mmol g^−1^ h^−1^ [126]. Although great achievements have been reached, the present H_2_ evolution rate of this kind of material is far from practical application. There are several strategies proposed to further improve the H_2_ evolution activity of halide perovskites: the first approach is the controllable synthesis of nanostructures with definite morphologies such as nanosheets, nanoplatelets to afford more exposed surfaces, and to increase the surface area and provide more active sites. Another option is the combination of single-atom catalysis with halide perovskites. Owing to the rapid increase of surface-free energy, quantum confinement effects, unsaturated coordination, and interactions between the metal with reduced size and substrate, the catalytic activity and stability of halide perovskites can be improved [43]. The construction of novel heterojunctions between halide perovskites and a suitable charge-transporting motif with desirable/well matching band alignment can enhance the charge separation [129]. For example, by coupling halide perovskites with electron- or hole-transporting materials such as GO, rGO, MXene, MOF, etc., can effectively promote charge separation and migration, thereby resulting in efficient catalytic activity. Type-II and Z-scheme are the most widely reported heterojunctions to achieve rapid charge transfer.

### 4.3. Enhance the Redox Ability of Halide Perovskites

The relatively narrow bandgaps of halide perovskites, which are especially associated with the VB edges, will inevitably bring about poor oxidation abilities. The weak oxidation capacity of halide perovskites will limit their application in some oxidation reactions—for instance, water oxidation (H_2_O/O_2_ at 1.23 eV vs. RHE) and organic compound mineralization (OH^−^/•OH at 1.67 eV vs. RHE). The combination of halide perovskites with other semiconductors with more positive VB (building a Z-scheme heterojunction) can achieve strong redox capabilities, broad light absorption, and efficient charge separation. Alternatively, by encapsulating the perovskite layer in a solar cell architecture to develop a perovskite-based PEC reaction system, the redox ability of perovskite can be enhanced by the applied voltage to achieve a broad reaction scope [43].

### 4.4. Exploring Mechanisms of Perovskite-Based Photocatalysis by Combining Experimental and Theoretical Research

Despite the great progress made in perovskite-based photocatalysis, there is a lack of comprehensive understanding of reaction mechanisms, such as the catalytic kinetic processes, the photophysical processes, and microscopic mechanisms of the involved surface chemical reactions. Hence, a complete theoretical model is required to interpret the roles of perovskite materials in photocatalytic redox reactions. Theoretical studies can not only help to enhance the understanding of established activities, but also can provide guidance for developing more efficient photocatalysts for redox reactions. By combining theoretical calculations and in-situ characterization techniques, mechanisms of perovskite-based photocatalysis such as reaction pathways and changes of catalysts during photocatalysis can be probed.

In this review, we introduced up-to-date progress of halide perovskites in photocatalytic hydrogen production. Up to this stage, perovskite powder (photocatalysis) and thin film (PEC and PV-PEC)-based photocatalysis systems have been proved to be effective for solar fuel production. However, from the viewpoint of commercialization, the factors of yield, reaction kinetics, stability, scalability, and cost and simplicity of production shall be reassessed. In addition, the general photocatalytic activity and stability of perovskite-based materials are currently far from optimal, and their application in photocatalysis is still in its infancy. The use of perovskites to address energy and environmental concerns still faces many challenges. These challenges also imply large opportunities for further exploration of perovskite-based photocatalysts with improved activity and more potential reactions. We hope that this review can provide some guidance toward finding optimal performance and stability for perovskite-based photocatalytic applications.

## Figures and Tables

**Table 1:** Summary of photocatalytic hydrogen evolution activity of halide perovskite-based systems.

Photocatalyst	Solution	Light Source	Activity (μmol g^−1^ h^−1^)	Stability (h)	Ref.
MAPbI_3_/Pt	Aqueous HI	visible light (λ ≥ 475 nm)	57	160	[44]
MAPbI_3−x_Br_x_/Pt	Aqueous HBr/HI	visible light (λ ≥ 420 nm)	2604	>30	[110]
Pt/TiO_2_-MAPbI_3_	Aqueous HI	visible light (λ ≥ 420 nm)	77.9	>12	[42]
MAPbI_3_/rGO	Aqueous HI	visible light (λ ≥ 420 nm)	939	200	[41]
BP/MAPbI_3_	Aqueous HI	visible light (λ ≥ 420 nm)	3472	200	[114]
PEDOT:PSS/MAPbBr_3_/Ta_2_O_5_	Aqueous HBr	visible light (λ ≥ 420 nm)	1050	>4	[115]
MAPbI_3_/MoS_2_	Aqueous HI	white LED lamp (450 nm)	1963	>24	[116]
MA_3_Bi_2_I_9_/Pt	Aqueous HI	visible light (λ ≥ 400 nm)	170	70	[117]
CsPbBr_3−x_I_x_/Pt	Aqueous HBr/HI	visible light (λ ≥ 420 nm)	1120	>50	[113]
Cs_2_AgBiBr_6_/rGO	Aqueous HBr	visible light (λ ≥ 420 nm)	48.9	120	[118]
DMASnI_3_	DI water	300 W Xe lamp (full spectrum)	3.2	>5	[119]
Ni_3_C/MAPbI_3_	Aqueous HI	visible light (λ ≥ 420 nm)	2362	>100	[121]
MAPbI_3_/CoP	Aqueous HI	visible light (λ ≥ 420 nm)	2087.5	27	[122]
Pt-DA_3_BiI_6_	Aqueous HI	100 W white LED lamp	91	>16	[123]
MA_3_Bi_2_I_9_/DMA_3_BiI_6_	Aqueous HI	visible light (λ ≥ 420 nm)	198.4	>90	[124]
MA^+^-crafted MAPbI_3_	Aqueous HI	visible light (λ ≥ 420 nm)	313	_	[125]
ML-MoS_2_/MAPbI_3_-MC	Aqueous HI	visible light (λ ≥ 420 nm)	13,600	208	[126]
MoS_2_/M_0.6_F_0.4_PbI_3_	Aqueous HI	visible light (λ ≥ 420 nm)	2131	>90	[127]
PtI_x_/[(CH_3_)_2_NH_2_]_3_[BiI_6_]	Aqueous HI	Commercial LED lamp, λ = 425 nm	94	100	[128]
